# Induction of maturation and activation of human dendritic cells: A mechanism underlying the beneficial effect of *Viscum album *as complimentary therapy in cancer

**DOI:** 10.1186/1471-2407-8-161

**Published:** 2008-06-04

**Authors:** Sri Ramulu Elluru, Jean-Paul Duong van Huyen, Sandrine Delignat, Michel D Kazatchkine, Alain Friboulet, Srini V Kaveri, Jagadeesh Bayry

**Affiliations:** 1 Centre de Recherche des Cordeliers, Université Pierre et Marie Curie – Paris6, UMR S 872, Paris, 75006, France; 2 Université Paris Descartes, UMR S 872, Paris, F-75006, France; 3 INSERM, U872, Paris, F-75006, France; 4 CNRS UMR6022, Université de Technologie, Compiègne, France; 5 Laboratoire d'Anatomie Pathologique, Hôpital Européen Georges Pompidou, 20-40 rue Leblanc, Paris, France

## Abstract

**Background:**

*Viscum album *(VA) preparations have been used as a complimentary therapy in cancer. In addition to their cytotoxic properties, they have also been shown to have immunostimulatory properties. In the present study, we examine the hypothesis that the VA preparations induce activation of human DC that facilitates effective tumor regression.

**Methods:**

Four day old monocyte-derived immature DCs were treated with VA Qu Spez at 5, 10 and 15 μg/ml for 48 hrs. The expression of surface molecules was analyzed by flow cytometry. The ability of Qu Spez-educated DC to stimulate T cells was analyzed by allogeneic mixed lymphocyte reaction and activation of Melan-A/MART-1-specific M77-80 CD8+T cells. Cytokines in cell free culture supernatant was analyzed by cytokine bead array assay.

**Results:**

VA Qu Spez stimulated DCs presented with increased expression of antigen presenting molecule HLA-DR and of co-stimulatory molecules CD40, CD80 and CD86. The VA Qu Spez also induced the secretion of inflammatory cytokines IL-6 and IL-8. Further, Qu Spez-educated DC stimulated CD4+T cells in a allogeneic mixed lymphocyte reaction and activated melanoma antigen Melan-A/MART-1-specific M77-80 CD8+T cells as evidenced by increased secretion of TNF-α and IFNγ.

**Conclusion:**

The VA preparations stimulate the maturation and activation of human DCs, which may facilitate anti-tumoral immune responses. These results should assist in understanding the immunostimulatory properties of VA preparations and improving the therapeutic strategies.

## Background

VA preparations are aqueous extracts from *Viscum album *(also known as European mistletoe) consisting of different types of lectins [1-3]. In addition to mistletoe lectins (ML), biologically active components of VA preparations include viscotoxins, several enzymes, peptides (such as viscumamide), amino acids, thiols, amines, polysaccharides, cyclitoles, lipids, phytosterols, triterpines, flavonoids, phenylpropanes and minerals [3,4]. VA preparations have been used as a complimentary therapy in cancer. Several studies have reported the clinical benefits of VA preparations in cancer patients [5,6]. Treatment with VA preparations or purified ML has also been shown to be associated with tumor regression in several experimental models [7,8]. The mechanisms underlying the anti-tumoral activity of VA preparations are complex and not completely understood. The proposed mechanisms include induction of apoptosis of tumor cells and lymphocytes, inhibition of angiogenesis and stimulation of the cellular compartment of the immune system [9-14].

During the course of tumor development, the tumor evades the immune system through the secretion of various factors such as VEGF, IL-10 and PGE_2 _that have been shown to inactivate the immune system [15]. The different pathways of immune evasion by tumors involve: induction of immune tolerance, resistance to killing by immune effector cells, and imparting functional paralysis of professional antigen presenting cells (APCs) such as dendritic cells (DCs) [15].

DCs are the professional APCs that are specialized in the uptake of antigens and their transport from peripheral tissues to the lymphoid organs [16,17]. Because of their capacity to stimulate naive T cells, DCs have a central role in the initiation of primary immune responses [18]. DCs reside in periphery as immature cells with a high ability to endocytose target antigens [19]. Upon receiving appropriate stimuli and in the context of inflammation, DCs undergo maturation process characterized by increased surface expression of antigen presenting HLA molecules and co-stimulatory molecules such as CD80 and CD86 and secrete several pro-inflammatory cytokines [20].

Tumor cells suppress the maturation and activation process of DCs [21]. Thus, several studies have demonstrated that DCs that reside in the tumor site or in the vicinity of tumor are immature with a decreased ability to stimulate T cells [22,23]. In addition, tumor cells secrete several anti-inflammatory cytokines such as IL-10 and TGF, which can suppress the functions of DCs [24-26]. In view of the anti-tumoral and immunostimulatory properties of VA preparations, and the central role of DCs in anti-tumoral immune response, we examined the hypothesis that VA preparations stimulate the DCs, which in part explains the mechanisms underlying the beneficial effect of VA preparations in cancer therapy.

## Methods

### Antibodies and reagents

Recombinant human (rh) interleukin-4 (IL-4) was obtained from R&D Systems (Lille, France), and rh granulocyte macrophage-colony-stimulating factor (rh GM-CSF), rhIL-2 and rhTNFα were obtained from Immunotools (Friesoythe, Germany). FITC-conjugated monoclonal antibodies (mAb) to HLA-DR and CD80, PE-conjugated mAbs to CD86, CD40 and CD83 and APC-conjugated mAbs to CD11c were obtained from BD biosciences (France).

### VA preparations

VA Qu Spez, was a kind gift from Weleda AG (Arlesheim, Switzerland). VA Qu Spez is the extract of *Viscum album *growing on oak trees. The VA preparations are therapeutic preparations that are free from endotoxins. VA preparations are formulated in sodium chloride (NaCl 0.9%) isotonic solution as 5 mg/ml vials. During the manufacturing process, VA preparations are prepared by standardizing the levels of Mistletoe lectins and Viscotoxins. The concentrations of the lectins and viscotoxins of the different preparations used in the study are summarized in Table 1.

**Table 1:** Concentrations of Mistletoe Lectins and Viscotoxins in VA Qu Spez

**Preparation**	**Concentration used (μg/ml)**	**Lectin (ng/ml)**	**Viscotoxin (ng/ml)**
VA Qu Spez	5	0.375	0.012
VA Qu Spez	10	0.750	0.024
VA Qu Spez	15	1.125	0.036

### Generation and culture of monocyte-derived human dendritic cells

Peripheral blood mononuclear cells (PBMC) were isolated from buffy coats of healthy donors purchased from Hopital Hotel Dieu, Etablissement Français du Sang (06/EFS/029, dated 29.05.2006), upon ethical approval for the use of such materials. The percentage of monocytes in the PBMC preparations was in the range of 9 to 14%. Monocytes were positively isolated using CD14 beads (Miltenyi Biotec, France). The purity of the monocytes after purification is > 98%. Immature DCs were generated by culturing monocytes for 4 days in RPMI 1640 containing 10% FCS, 50 U/ml penicillin, 50 μg/ml streptomycin, rhIL-4 (500 IU/10^6 ^cells), and rhGM-CSF (1000 IU/10^6 ^cells). Half of the medium, including all supplements, was replaced on second day.

### Analysis of the expression of surface molecules by flow cytometry

To investigate the effect of VA Qu Spez on DCs, 0.5 × 10^6 ^immature four-day old DCs were either untreated or treated with TNFα (15 ng) or VA preparations (5, 10 and 15 μg) for 48 h. On day 6, cell surface staining was performed with specifically labeled mAbs and proceeded for flow-cytometry (LSR II, BD Biosciences, France). Ten thousand events were recorded and analyzed for each sample. Data were analyzed by BD FACSDIVA software (BD Biosciences, France).

### Mixed lymphocyte reaction (MLR) with allogenic CD4+ T cells

Responder CD4+ T cells used for allogeneic MLR assays were isolated from PBMC of healthy donors using a negative isolation kit (Dynal biotech-Invitrogen, France). DCs following 48 hr treatment with VA Qu Spez were washed extensively and were seeded with 1 × 10^5 ^responder allogeneic T cells at DC:T cell ratios of 1:10, 1:20 and 1:40. After 4 days, the cells were pulsed for 16 h with 0.5 μCi (0.037 MBq) of (^3^H)thymidine. Radioactive incorporation was measured by standard liquid scintillation counting. The proliferation of cells was measured as counts per minute (mean ± SEM of triplicate values) after subtracting values of responder T cell cultures alone.

### Anergy assay to determine the activation status of the CD4 T cells in the co-culture with VA-treated DCs

The anergy assay was performed according to a modified protocol originally described by Steinbrink et al [27]. Briefly, four-day old DCs were treated for 48 hrs with VA Qu Spez (15 μg/ml) or untreated or TNFα (15 ng/ml). Responder CD4+ T cells were then co-cultured during the first incubation at a density of 10^5 ^cells with 10^4 ^DC for 72 hrs. Then, T cells from the co-cultures were isolated by using CD4+ beads (Miltenyl Biotech) and rested for 24 hrs in the culture medium containing 2 U/ml IL-2. Subsequently, CD4+ T cells were re-stimulated with DCs generated from the same donor as that used for the first stimulation and have undergone similar VA Qu Spez treatment. After 48 hrs, the cells were pulsed for 16 h with 0.5 μCi (0.037 MBq) of (^3^H)thymidine. Radioactive incorporation was measured by standard liquid scintillation counting. The proliferation of cells was measured as counts per minute (mean ± SEM of triplicate values). Tests were conducted in triplicates. Additionally, the levels of cytokines TNF-α and IFNγ in the co-culture were analysed.

### Activation of melanoma specific cytotoxic T cell (CTL) clones by VA Qu Spez-treated DCs

The melan-A-specific CTL clone M77-80 that was derived from tumor infiltrating lymphocytes of melanoma patient M77 is a kind gift from Dr. Nathalie Labarriere and Dr. Francine Jotereau [28,29]. The VA Qu Spez-treated DCs from HLA matched donor (HLA-A2, 10^4^/well/200 μl RPMI 1640 medium supplemented with 10% AB serum) were cultured overnight with M77 CTLs (10^5^) in 96 well round-bottomed plates along with the MART-1 peptide (1 μM) and 25 IU/mL rh IL-2. The activation of M77-80 was analyzed by measuring IFNγ and TNFα in the cell free-supernatants.

### Analysis of cytokines

Cytokines in the cell-free culture supernatant were quantified using BD CBA Human Inflammation kit and Human Th1/Th2 kits (BD Biosciences, France).

### Statistical analysis

Statistical significance was determined using the Mann-Whitney U test.

## Results

### VA Qu Spez enhances the expression of antigen presenting and co-stimulatory molecules on human DCs

**Figure 1:**
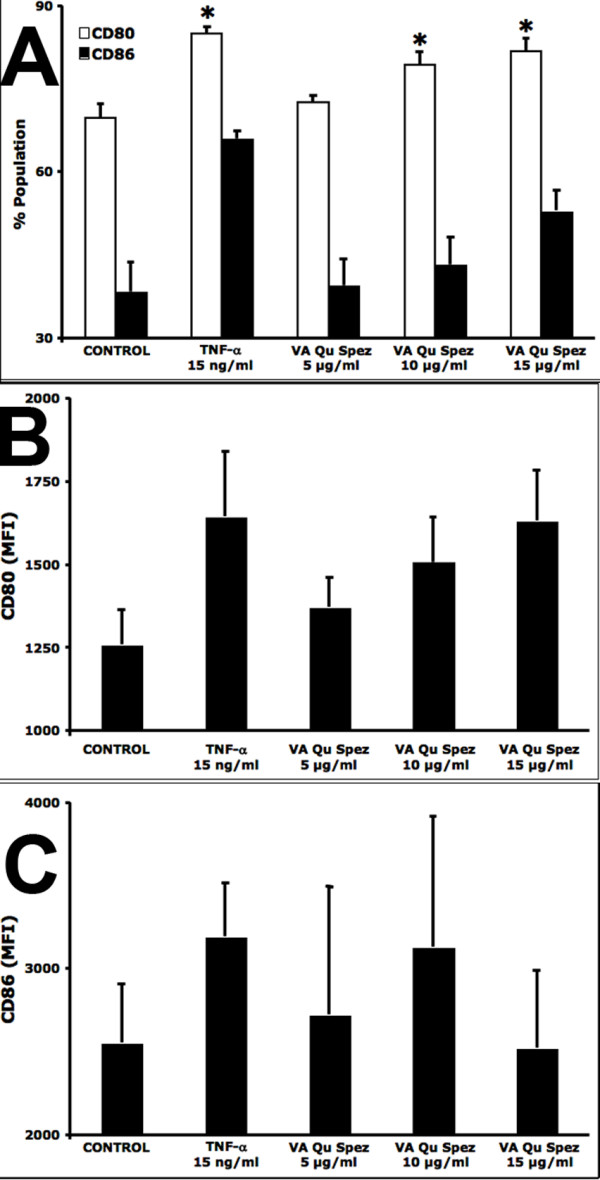
**DCs treated with VA Qu Spez show an increased expression of co-stimulatory molecules CD80 and CD86**. The 4-day-old DCs (0.5 × 10^6^) were treated with medium alone (Control) or with 15 ng/ml TNF-α or with 5, 10 and 15 μg/ml of VA Qu Spez for 48 hours. The expression of CD80 (A and B) and CD86 (A and C) was analysed by flow cytometry (BD LSRII). Panel A shows the % of DCs positive for CD80 (open bars) and CD86 (filled bars), while mean fluorescence intensities were presented in Panels B and C. Data are presented as mean ± SEM from from five to six independent donors. Statistical significance (*, p < 0.05) as analysed by Mann-Whitney test is indicated.

We initially characterized the effect of VA Qu Spez on the phenotype of human DCs. Four-day old immature DCs were treated with VA preparations for 48 hrs and cells were analyzed for the expression of various surface molecules (Figure 1). We have used DCs treated with 15 ng/ml of TNF-α as control in addition to the DCs that were left untreated. VA Qu Spez enhanced the expression by DCs of co-stimulatory molecules CD80 and CD86 (Figure 1) in dose-dependent manner. The expression of CD80 on DCs by 15 μg/ml concentration of VA Qu Spez (81.74 ± 2.3% population and 1632.8 ± 152 mean fluorescence intensity, MFI) was comparable to the DCs treated with TNF-α (84.93 ± 1.1% and 1644.25 ± 195.4 MFI). In addition, Qu Spez also significantly enhanced the percentage expression CD86 in a dose-dependent manner (Figure 1). However, the expression of HLA-DR, CD40 and CD83 were either unaltered or marginally increased (data not shown).

### VA Qu Spez induces the secretion of pro-inflammatory cytokines IL-6 and IL-8 by DCs

**Figure 2:**
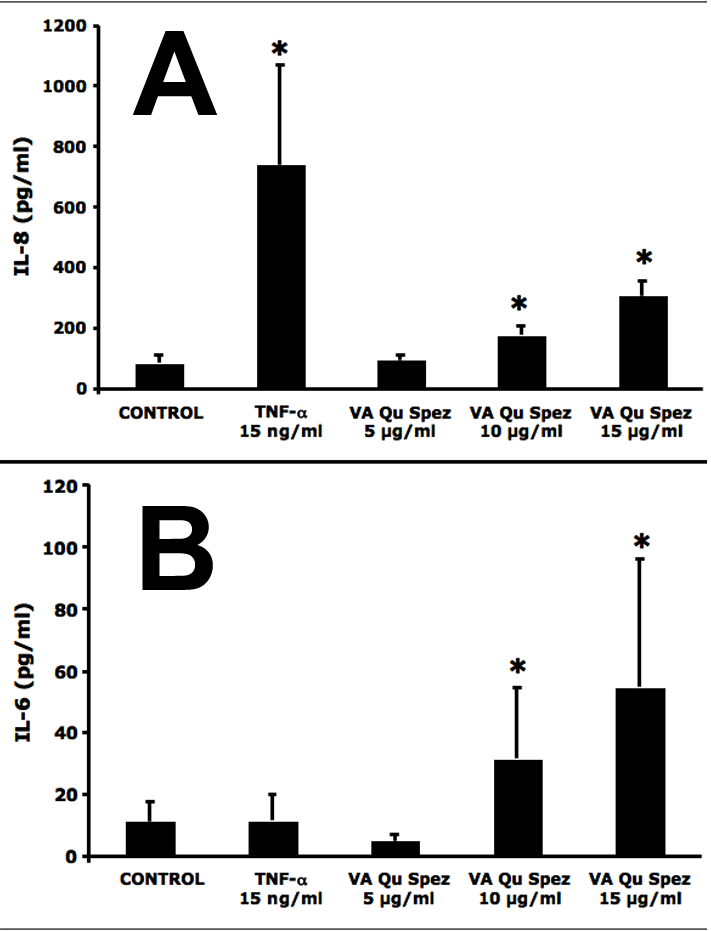
**DCs treated with VA Qu Spez show an increased secretion of inflammatory cytokines IL-8 and IL-6**. The 4-day-old DCs (0.5 × 10^6^) were treated with medium alone (Control) or with 15 ng/ml TNF-α or with 5, 10 and 15 μg/ml of VA Qu Spez for 48 hours. The secretion (pg/ml) of IL-8 (Panel A) and IL-6 (Panel B) in cell free supernatants were analysed by cytokine bead array assay. Data are presented as mean ± SEM from five to six independent donors. Statistical significance (*, p < 0.05) as analysed by Mann-Whitney test is indicated.

In addition to co-stimulatory molecules, DC-derived cytokines play a crucial role in priming T-cell response. We therefore analyzed whether the maturation process of DCs induced by VA Qu Spez is associated with the secretion of pro-inflammatory cytokines such as IL-6 and IL-8. The control DCs secreted 86.02 ± 23.3 pg/ml of IL-8 and 11.55 ± 6.28 pg/ml of IL-6 (n = 5 donors). However, as shown in Figure 2, VA Qu Spez significantly stimulated the secretion of IL-8 (308.052 ± 48.60 pg/ml) and IL-6 (54.97 ± 41.27 pg/ml) by DCs. Together these results indicate that in addition to stimulating the expression of co-stimulatory molecules on DCs, VA Qu Spez induce the secretion of pro-inflammatory cytokines.

### VA Qu Spez-treated DCs stimulate T cell proliferation

**Figure 3:**
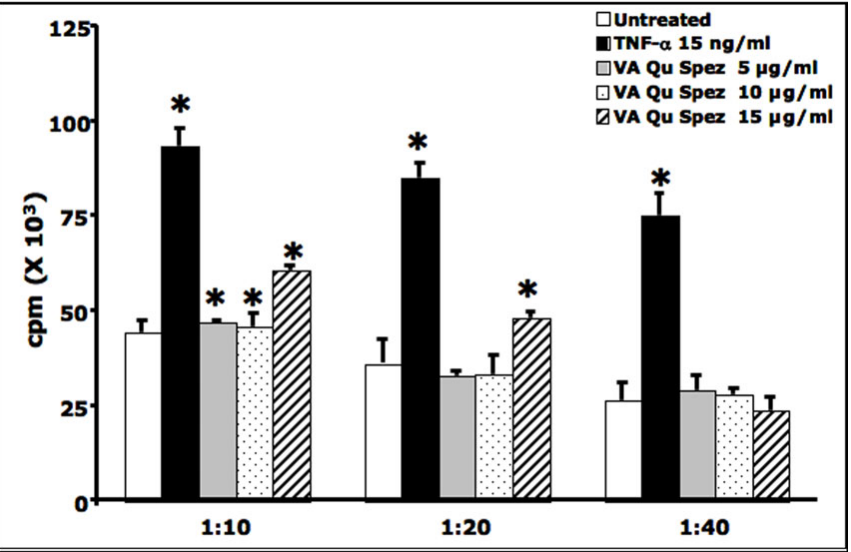
**VA Qu Spez-treated DCs stimulate the proliferation of allogeneic CD4+ T cells**. The 4-day-old DCs (0.5 × 10^6^) were treated with medium alone (Control) or with 15 ng/ml TNF-α or with 5, 10 and 15 μg/ml of VA Qu Spez for 48 hours. After 48 hr treatments, DCs were co-cultured with the allogeneic CD4+ T cells at different ratios in a round bottom 96-welled plate. After 4 days of co-culture, the cells were pulsed overnight with 0.5 μCi (0.037 MBq) of (^3^H)thymidine to quantify T-cell proliferation. Radioactive incorporation was measured by standard liquid scintillation counting, and the results were expressed as counts per minute (mean ± SEM of triplicate values). Statistical significance (*, p < 0.05) as analysed by Mann-Whitney test is indicated.

A major function of DCs is their ability to trigger the activation and proliferation of T cells. We thus examined whether maturation of DCs induced by VA Qu Spez is reflected in their capacity to stimulate CD4+ T cells in an allogeneic MLR. As shown in Figure 3, VA Qu Spez-treated DCs, stimulated the proliferation of CD4+ T cells in a dose-dependent manner. The extent of CD4+ T cell proliferation induced by VA Qu Spez-treated DCs was significant at DC-T cell ratios of 1:10 and 1:20.

### VA Qu Spez-treated DCs do not induce anergy of CD4+ T cells

**Figure 4:**
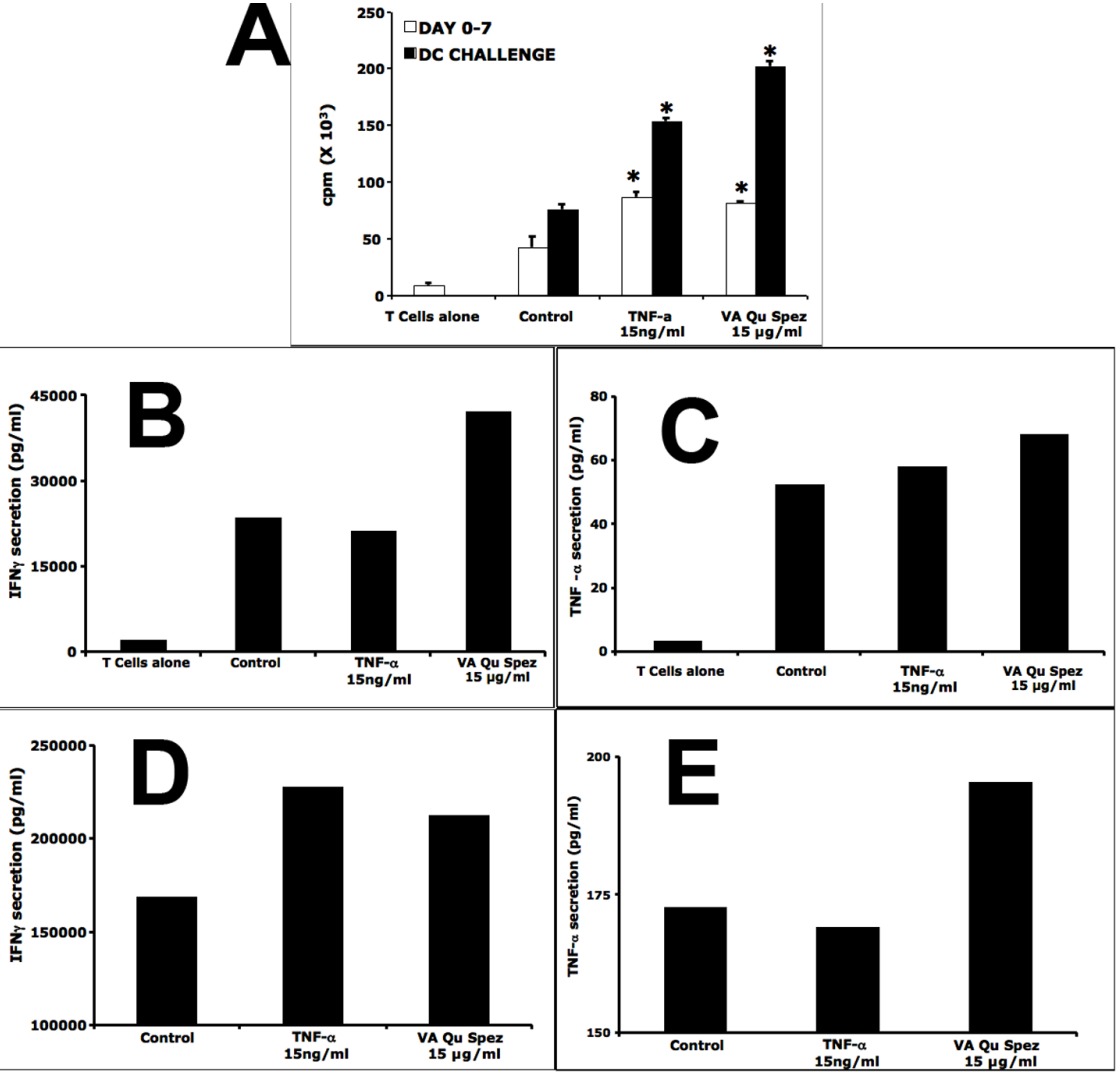
**VA Qu Spez-treated DCs do not induce CD4+ T cell anergy**. The 4-day-old DCs (0.5 × 10^6^) were treated with medium alone (Control) or with 15 ng/ml TNF-α or with 15 μg/ml of VA Qu Spez for 48 hours. The DCs were then co-cultured with the allogeneic CD4+ T cells at 1:10 ratio in a round bottom 96-welled plate for 72 hrs for the first cycle of stimulation. The T cells from in the co-cultures were then purified and were rested for 24 hrs in the presence of 2 IU/ml of IL-2. These CD4+ T cells were then subjected for second cycle of stimulation with similiarly treated DCs from same donor. After 48 hrs of co-culture, the cells were pulsed overnight with 0.5 μCi (0.037 MBq) of (^3^H)thymidine to quantify T-cell proliferation (Panel A, filled bars). Radioactive incorporation was measured by standard liquid scintillation counting, and the results were expressed as counts per minute (mean ± SEM of triplicate values). DC-T cell co-cultures of first cycle of stimulation that were maintained for 7 days were used for the comparison (Panel A, open bars). Statistical significance (*, p < 0.05) as analysed by Mann-Whitney test is indicated. The level of T cell cytokines IFNγ (Panels B and D) and TNF-α (Panels C and E) in the cell-free supernatants from above cultures were analysed by cytokine bead array. Panels B and C indicate the level of cytokines in DC-T cell co-cultures of first cycle of stimulation that were maintained for 7 days. Panels D and E present the level of cytokines in DC-T cell co-cultures of second cycle of stimulation.

We performed anergy assay to determine the activation status of the CD4 T cells in the co-culture with VA Qu Spez-treated DCs. As shown in Figure 4a, during first cycle of CD4+ T cell stimulation in a MLR (day 0–day 7), VA Qu Spez-treated DCs (81920 ± 9070 cpm) show similar ability to stimulate the proliferation of CD4+ T cells as compared to TNF-α treated DCs (87439 ± 3910 cpm). Interestingly, CD4+ T cells that were re-stimulated/challenged during second cycle of stimulation with VA Qu Spez-treated DCs show increased proliferation (202276 ± 2052 cpm) as compared to the control DCs (76236 ± 4436 cpm) and TNF-α treated DCs (154341 ± 3224 cpm).

To further confirm that VA Qu Spez-treated DCs do not impart CD4+T cell anergy, we analysed for the secretion of T cell cytokines TNF-α and IFNγ in the DC-CD4+T cell co-cultures. As shown in Figure 4b, CD4+ T cells that were re-stimulated/challenged during second cycle of stimulation with VA Qu Spez-treated DCs show increased secretion of above cytokines as compared to control DCs. These results suggest that maturation and activation of DCs induced by VA Qu Spez have functional repercussion on T cell activation and not T cell anergy.

### VA Qu Spez-treated DCs stimulate melanoma specific M77-80 CTL clone

**Figure 5:**
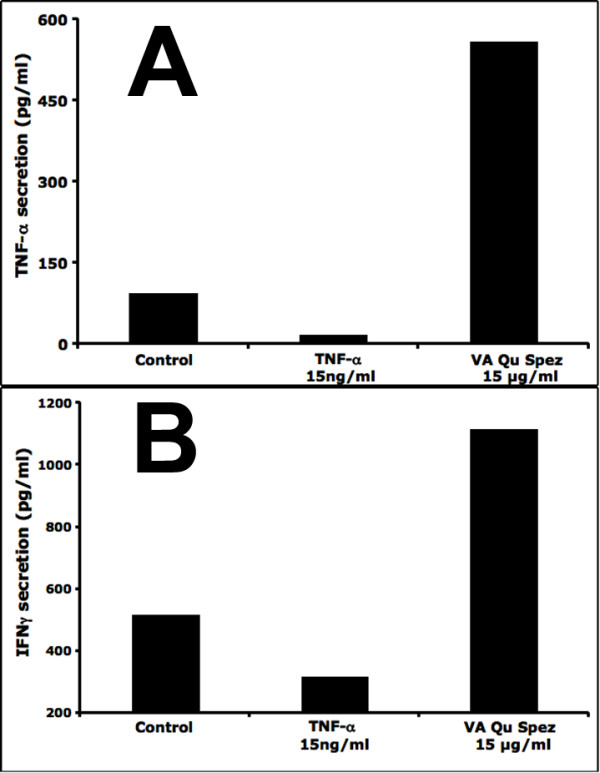
**VA Qu Spez-treated DCs stimulate melanoma specific M77-80 CTL clone**. The 4-day-old DCs (0.5 × 10^6^) from HLA-A2 donor were treated with medium alone (Control) or with 15 ng/ml TNF-α or with 15 μg/ml of VA Qu Spez for 48 hours. The DCs (10^4^/well/200 μl medium) were then cultured overnight with M77-80 CTLs (10^5^) in 96 well round-bottomed plates along with the MART-1 peptide (1 μM) and 25 IU/mL rh IL-2. The activation of M77-80 was analyzed by measuring TNFα (Panel A) and IFNγ (Panel B) in the cell free-supernatants by using cytokine bead array.

Since, VA preparations have been used as a complimentary therapy in cancer we examined whether VA Qu Spez-stimulated DCs facilitate anti-tumoral T cell responses. Therefore, HLA-matched VA Qu Spez-treated DCs were co-cultured with Melan-A/MART-1 specific M77-80 CTL clone (Figure 5). Strikingly, VA Qu Spez-treated DCs activated tumor antigen specific CTL clone as analyzed by the secretion of cytokines TNF-α and IFNγ.

## Discussion

Although VA preparations are widely used in clinical practice and cancer therapy, their mechanisms of action are yet to be fully understood. In our previous studies, we have shown that in addition to cytotoxic properties, VA preparations have immunostimulatory effects that facilitate tumor regression in experimental models [13]. However, to mount an effective anti-tumoral immune response, an increased expression of co-stimulatory molecules on the DCs, the sentinels of the immune system, accompanied by an enhanced secretion of pro-inflammatory cytokines that culminates in T cell proliferation is necessary.

DCs found within the tumor microenvironment are found to have a relatively immature phenotype characterized by low levels CD86, and surface HLA-DR expression and inability to produce pro-inflammatory cytokine [30,31]. Clinical studies with mistletoe lectins have shown that VA preparations stimulate the cytokine secretion and function of monocytes, the precursors of DCs [32]. The previous studies by Stein et al demonstrated that mistletoe extract and their isolated components influences the maturation of DC with an increased expression of co-stimulatory and antigen presenting molecules [33,34]. Furthermore, we found that the up-regulation of these molecules is accompanied by the induction of inflammatory cytokines by the VA preparations and stimulation of tumor specific T cells. Together these results suggest that induction of maturation and activation of human DCs is one of the mechanisms underlying the beneficial effect of VA preparations as complimentary therapy in cancer.

Previously, it has been demonstrated that VA lectin induces the gene expression of IL-1 alpha, IL-1 beta, IL-6, TNF-α, IFN-γ and GM-CSF from PBMC [35]. A recent clinical study has shown that the CD14+ monocytes from multiple myeloma patients could be induced to differentiate into functional DCs by culturing them with the cytokine cocktail consisting of GM-CSF, IL-4, IL-6, TNF-α and IL-1β for use in cancer immunotherapy [36]. Our data demonstrates that VA Qu Spez-mediated maturation of DCs and secretion of pro-inflammatory cytokines (IL-6 and IL-8) has repercussion on the stimulation of CD4+ T cells and their cytokine secretion. It is interesting to note that VA Qu Spez-treated DCs do not induce anergy of T cells as shown by the induction of proliferation and the secretion of TNF-α and IFNγ by the CD4+ T cells. Thus, induction of DC-cytokines and T cell cytokines by VA Qu Spez represents a critical determinant in the development of effective innate immune responses against the tumor cells [37].

CD8^+ ^cytotoxic T lymphocytes (CTLs) are critical for the elimination of tumor. Thus, therapies aimed at expansion of CTLs and their functions holds the key in mounting an effective anti-tumor immune response. The ability of the CTLs to recognize the processed peptides derived from the cellular genes, such as those encoding MART-1 or tyrosinase in melanoma, led to the recognition that protective immune responses are often directed towards tumor-associated, rather than tumor-specific, antigens [28,29]. Using Melan-A/MART-1 specific M77-80 CTL clone, we have shown that DCs "educated" by VA Qu Spez can mount an anti-tumoral immune response as suggested by the increased levels of secretion of TNF-α and IFNγ by the CTLs in the co-culture. Further studies on the effect of the VA preparations on the DCs that have been subjected to inactivation by tumor factors, may provide strategies in dissecting the stimulatory effects of the VA preparations on the DCs.

## Conclusion

VA preparations are known to have cytotoxic properties towards the tumor cells. They are also known to improve the quality of life in the cancer patients. However, the mechanisms by which VA preparations stimulate the immune system and exert beneficial effects in patients are not yet clear. We have demonstrated the role of the VA preparations in stimulating the DCs with implications in the induction of anti-tumor immunity. However, these *in vitro *results need to be validated further in the context of clinical studies. The elucidation of immunostimulatory mechanisms of VA preparations is critical in understanding their role as complimentary therapy in cancer.

## Competing interests

This work was supported by research grant from Weleda AG. The authors declare that they have no competing interests.

## Authors' contributions

SE, SVK and JB participated in the study design. SE, JP-DVH and SD performed experiments. SE, MDK, AF, SVK and JB analysed the data. SE, SVK and JB prepared the manuscript. All authors read and approved the final manuscript.

## Pre-publication history

The pre-publication history for this paper can be accessed here:


